# Understanding the Limitations of Chloride Double Perovskites as Hosts for Stable Cr^3+^ Luminescence

**DOI:** 10.1021/acs.inorgchem.5c02293

**Published:** 2025-07-16

**Authors:** Kuan-Yi Lee, Hsiu-Kai Yang, Tadeusz Leśniewski, Mikołaj Kamiński, Natalia Majewska, Jakub Gnyp, Wei-Lun Su, Yi-Ting Tsai, Sebastian Mahlik, Mu-Huai Fang

**Affiliations:** † Research Center for Applied Sciences, 38017 Academia Sinica, Taipei 11529, Taiwan; ‡ Institute of Experimental Physics, Faculty of Mathematics, Physics and Informatics, 49646 University of Gdansk, Wita Stwosza 57, Gdansk 80-308, Poland; § Faculty of Physics, Kazimierz Wielki University in Bydgoszcz, Powstańców Wielkopolskich 2, Bydgoszcz 85-090, Poland; ∥ Faculty of Chemistry, Adam Mickiewicz University, Uniwersytetu Poznańskiego 8, Poznań 61-614, Poland

## Abstract

Chloride double perovskite, a promising frontier for cutting-edge light-conversion research worldwide, is harnessed by incorporating transition-metal ions and advanced crystal engineering. Despite the significant progress in transition-metal-ion-doped chloride double perovskite, challenges such as insufficient thermal stability remain substantial barriers to their practical applications. Additionally, the quenching mechanisms in these materials are not fully understood. This study presents a proof of concept by synthesizing Cr-doped Cs_2_AgInCl_6_ using a sintering-free ball-mill mechanochemistry method. The resulting Cs_2_AgIn_1–*x*_Cr_*x*_Cl_6_ exhibits an emission spectrum ranging from 850 to 1350 nm, bridging the gap between near-infrared and shortwave infrared regions. We thoroughly investigate the changes in the absorption coefficient, thermal quenching, and thermal-activated energy transfer using temperature-dependent photoluminescence and time-resolved spectra. Moreover, we utilize the photocurrent excitation (PCE) spectra to elucidate the quenching process and the associated energy states. Our findings reveal an unexpected luminescence quenching in the materials in the room temperature region, which is attributed to interactions between the Cr-excited states and self-trapped exciton (STE) states. This study explores how PCE can provide insights into the relationship between band structure and quenching dynamics, offering a perspective on the challenges of achieving efficient and thermally stable Cr^3+^ luminescence in chloride double perovskites.

## Graphical abstract

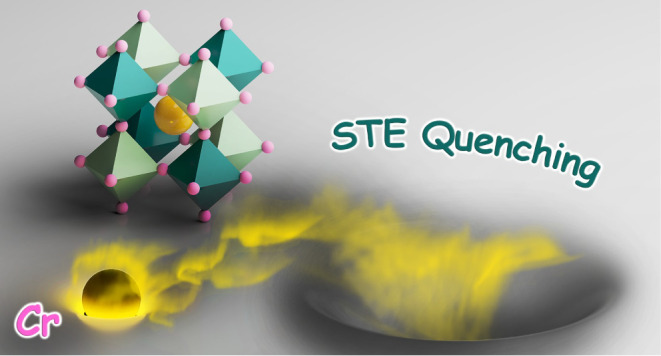

## Introduction

Halide perovskite materials have demonstrated significant potential in various advanced technologies, including light-emitting diodes,^1–3^ solar cells,^4,5^ photodetector,^6,7^ and laser.^8,9^ Recently, halide double-perovskites, with the structure of *A*_2_ *B’B″X*_6_, have garnered considerable attention due to their diverse crystal structures compared to the traditional *ABX*_3_ halide perovskites. These halide double-perovskites can be synthesized into different forms, such as nanocrystals,^10^ nanowires,^11^ two-dimensional materials,^12,13^ and three-dimensional bulk materials.^14^ Furthermore, the *A* site in these structures can be substituted with organic ligands to form hybrid double perovskite materials.^15^ These unique properties make halide double-perovskite an appealing option for various applications.

One of the emerging applications for halide double-perovskite is their integration into light-emitting diodes by incorporating luminescent activators into their crystal structure. While significant advancements have been made in luminescent materials operating in visible wavelength regions, those functioning in the near-infrared and shortwave infrared regions remain comparatively underdeveloped. Inorganic materials doped with transition metal ions (e.g., Ni^2+^, Cr^3+^/Cr^4+^, Mn^2+^) are at the forefront of pioneering broadband NIR and SWIR emission.^16–23^ When integrated into light-emitting diodes (pc-LEDs), these materials usher in an era of energy-efficient broadband light sources that operate with minimal heat generation. However, a notable challenge lies in the spectral range; most are optimized to emit either in the NIR spectrum (650–1000 nm) or the SWIR spectrum (1000–1500 nm), leading to a spectral gap between 900–1100 nm.^24,25^ Consequently, there is a pressing need for luminescent materials to bridge this gap, with an emission maximum peak around ∼1000 nm. In this context, Cr-doped Cs_2_AgInCl_6_ chloride double perovskite has garnered significant interest for its potential to emit at approximately 1000 nm, presenting a promising solution to this spectral discontinuity. Zhao et al.^26^ successfully synthesized the Cs_2_AgInCl_6_:Cr^3+^ powder by high-temperature solid-state reaction, in which the emission covers 850–1350 nm with the emission peak maximum at 1010 nm and full width at half-maximum (*fwhm*) of 180 nm. The thermal quenching temperature, denoted as *T*_50_ (defined as the emission intensity being 50% of its intensity at 80 K), was determined to be 320 K. Meanwhile, Chen et al. produced the Cs_2_AgInCl_6_:Cr^3+^/Mn^2+^ and Cs_2_AgInCl_6_:Cr^3+^/Yb^3+^ single crystals by hydrothermal methods. Notably, the *T*_50_ value was elevated from 320 to 350 K through Yb^3+^ doping.^27,28^ Gan et al. successfully prepared the Cs_2_AgInCl_6_:Cr^3+^/Er^3+^ using a coprecipitation method.^29^ Liu et al. fabricated the Cs_2_AgInCl_6_:Bi^3+^ nanocrystals through the hot injection method.^30^ Despite these advancements, these transition-metal-ion-doped chloride double perovskite materials exhibit much weaker luminescent intensity at room temperature compared to cryogenic temperatures, indicating significant thermal quenching, such as the *T*_50_ was calculated to be 200 K in Cs_2_NaScCl_6_:Cr^3+^.^26,29,31–34^ According to the literature above, chloride-based double perovskites typically exhibit stronger thermal quenching. Although this quenching phenomenon is known, the underlying mechanism remains ambiguous, highlighting the urgent need to develop techniques to comprehend and mitigate this issue.

In this study, as a proof-of-concept, we synthesized Cr-doped Cs_2_AgInCl_6_ using a sintering-free ball-mill mechanochemistry approach, marking the first application of this technique to Cr-doped Cs_2_AgInCl_6_. To address the challenges above, we thoroughly characterize the absorption coefficient, the thermal quenching, and thermal-activated energy transfer through temperature-dependent photoluminescence and time-resolved spectroscopy. Additionally, we exploit the photocurrent excitation (PCE) technique to investigate the electronic transition and the corresponding quenching mechanism, particularly the interaction between the excited state and the self-trapped exciton (STE) state. This study provides valuable insights into the quenching mechanisms and contributes to developing luminescent materials with suitable hosts and activators.

## Results and Discussion

### Structural Properties

The synchrotron X-ray diffraction (XRD) with the X-ray wavelength of 0.61992 Å is utilized to investigate the crystal structures of Cs_2_AgIn_1–*x*_Cr_*x*_Cl_6_ (*x* = 0.00, 0.01, 0.05, and 0.10), as shown in Figure 1a. The diffraction patterns fit well with the standard pattern derived from the crystallographic information framework (CIF) in the inorganic crystal structure database (ICSD) database with ICSD 244519.^35^ The *fwhm* of the diffraction peaks from Cs_2_AgIn_1–*x*_Cr_*x*_Cl_6_ is noticeably broader than that of materials synthesized through sintering. For instance, the *fwhm* of the XRD peak at 9.597° of Cs_2_AgIn_1–*x*_Cr_*x*_Cl_6_ is 0.163°, whereas the peak at 9.216° for LiGaO_2_:Cr, under the same conditions, is only 0.015°.^36^ This broader *fwhm* indicates small crystallites in the materials synthesized by the ball-mill method, which is attributed to the absence of the heating and sintering processes. A minimal presence of AgCl impurity exists in the samples, marked by an asterisk in Figure 1a. The incorporation of Cr^3+^ into the In site is expected, given the same oxidation states of Cr^3+^ and In^3+^, as well as the similar sizes of Cr^3+^ (0.615 Å; CN = 6) (CN—coordinated number) and In^3+^ (0.800 Å; CN = 6), compared to the Ag^+^ (1.15 Å; CN = 6).^37^ As the concentration of Cr^3+^ increases, the diffraction peaks shift to the larger angles due to the smaller ionic size of Cr^3+^ relative to In^3+^. Cs_2_AgInCl_6_ crystallizes in a cubic system with the space group of Fm3̅m , where both the In^3+^ and Ag^+^ ions are coordinated with six Cl^–^ ions, forming [InCl_6_] and [AgCl_6_] octahedrons, respectively, as depicted in Figure 1b. Furthermore, Cs_2_AgInCl_6_ consists of a three-dimensional (3D) network of ordered vertices-sharing [InCl_6_] and [AgCl_6_] octahedrons. Conversely, the Cs^+^ ions occupy the centers of the octahedrons, coordinated by 12 Cl^–^ ions.

**Figure 1:**
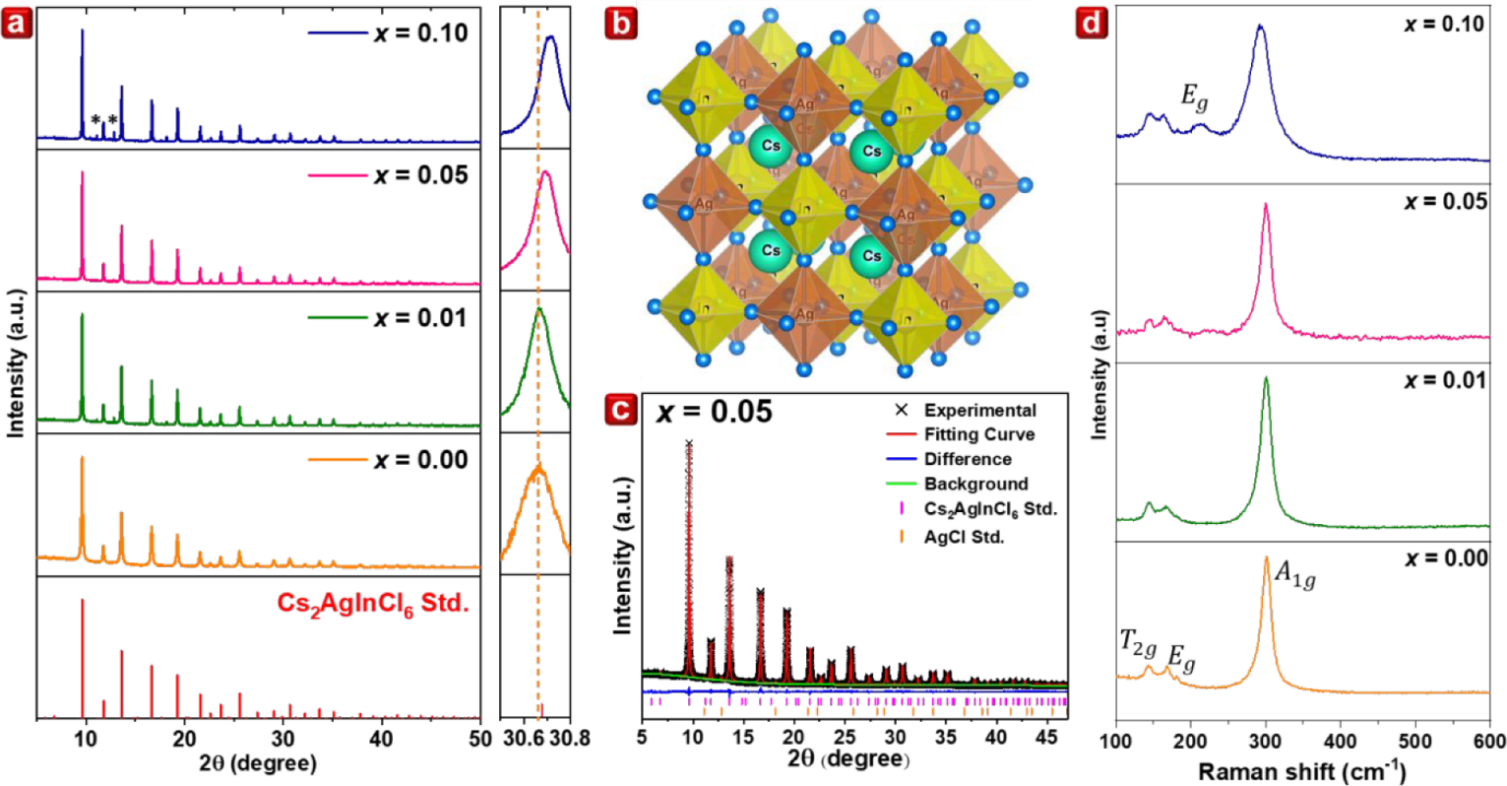
(a) Synchrotron XRD of Cs_2_AgIn_1–*x*_Cr_*x*_Cl_6_ with *x* = 0.00, 0.01, 0.05, and 0.10. The asterisk in the figure indicates the existence of the AgCl impurity. (b) Crystal structure of Cs_2_AgInCl_6_. (c) Rietveld refinement of Cs_2_AgIn_0.95_Cr_0.05_Cl_6_. (d) Raman spectra of Cs_2_AgIn_1–*x*_Cr_*x*_Cl_6_ with *x* = 0.00, 0.01, 0.05, and 0.10.

To elucidate the structural information after doping the Cr^3+^ ion, the Rietveld refinement of *x* = 0.05 is conducted, as shown in Figure 1c. Similar refinements of *x* = 0.00, 0.01, and 0.10 are depicted in Figure S1a–c. The results of these refinements, including details on atomic positions, atomic displacement parameters, occupancy values, atomic displacements, and other refined parameters of Cs_2_AgIn_1–*x*_Cr_*x*_Cl_6_ (*x* = 0.00, 0.01, 0.05, and 0.10), are listed in Tables S1 and S2. Given its detectable concentration level, the Cr ion is explicitly accounted for in the refinement model for samples with *x* = 0.05 and 0.10. The unit cell volume for *x* = 0.00, 0.01, 0.05, and 0.10 decreases linearly, as depicted in Figure S2, indicating a smooth integration of Cr^3+^ into the Cs_2_AgInCl_6_ host. The presence of AgCl impurity accounted for in the refinement was less than 2 wt % across all samples, as detailed in Table S2.

To investigate structural symmetry, Raman spectroscopy was performed on Cs_2_AgIn_1–*x*_Cr_*x*_Cl_6_ with varying Cr concentrations (*x* = 0.00, 0.01, 0.05, and 0.10), as shown in Figure 1d. The analysis revealed three distinct peaks at 143.4, 168.0, and 301.5 cm^–1^, consistent with previously reported data.^38^ According to literature, Cs_2_AgInCl_6_ is expected to exhibit four Raman active modes: 2*T*_2*g*_ + 1*E*_*g*_ + *A*_1*g*_.^39^ The *T*_2*g*_ with the lowest frequency (∼50 cm^–1^) corresponds to the translation of the Cs atom, but this frequency is too low to be detected in our system.^40^ In contrast, the 1*E*_*g*_ and *A*_1*g*_ are associated with the stretching vibrations of the octahedrons.^40^ Interestingly, an additional peak at 213.0 cm^–1^ appears in the Raman spectra of *x* = 0.05 and 0.10 samples, likely from the CrCl_6_ octahedron with *E*_*g*_ mode.^40^ Furthermore, the [AgCl_6_] and [InCl_6_] octahedrons contribute to the *A*_1*g*_ mode, and after the incorporation of the Cr^3+^, the [CrCl_6_] octahedron also contributes to the *A*_1*g*_ mode, resulting in the broadening of the *A*_1*g*_ peak.^40^

### Morphology

The morphological properties of the phosphor materials are critically evaluated using optical microscopy (OM). Images of Cs_2_AgIn_1–*x*_Cr_*x*_Cl_6_ (*x* = 0.00, 0.01, 0.05, and 0.10) were captured at magnifications of 1500× (Figure 2a–d) and 150× (Figure S3a–d). OM provides information about the powder color and material transparency. Samples with Cr concentrations of *x* = 0.00, 0.01, and 0.05 exhibited white to light gray, while the *x* = 0.10 samples displayed light purple. At the higher magnification of 1500×, the transparent morphologies of the *x* = 0.00, 0.01, and 0.05 samples were evident, aligning with the typical morphological features of halide-based materials. However, the *x* = 0.10 samples showed reduced transparency, attributable to the absorption properties of Cr^3+^ within the Cs_2_AgInCl_6_ host matrix.

**Figure 2:**
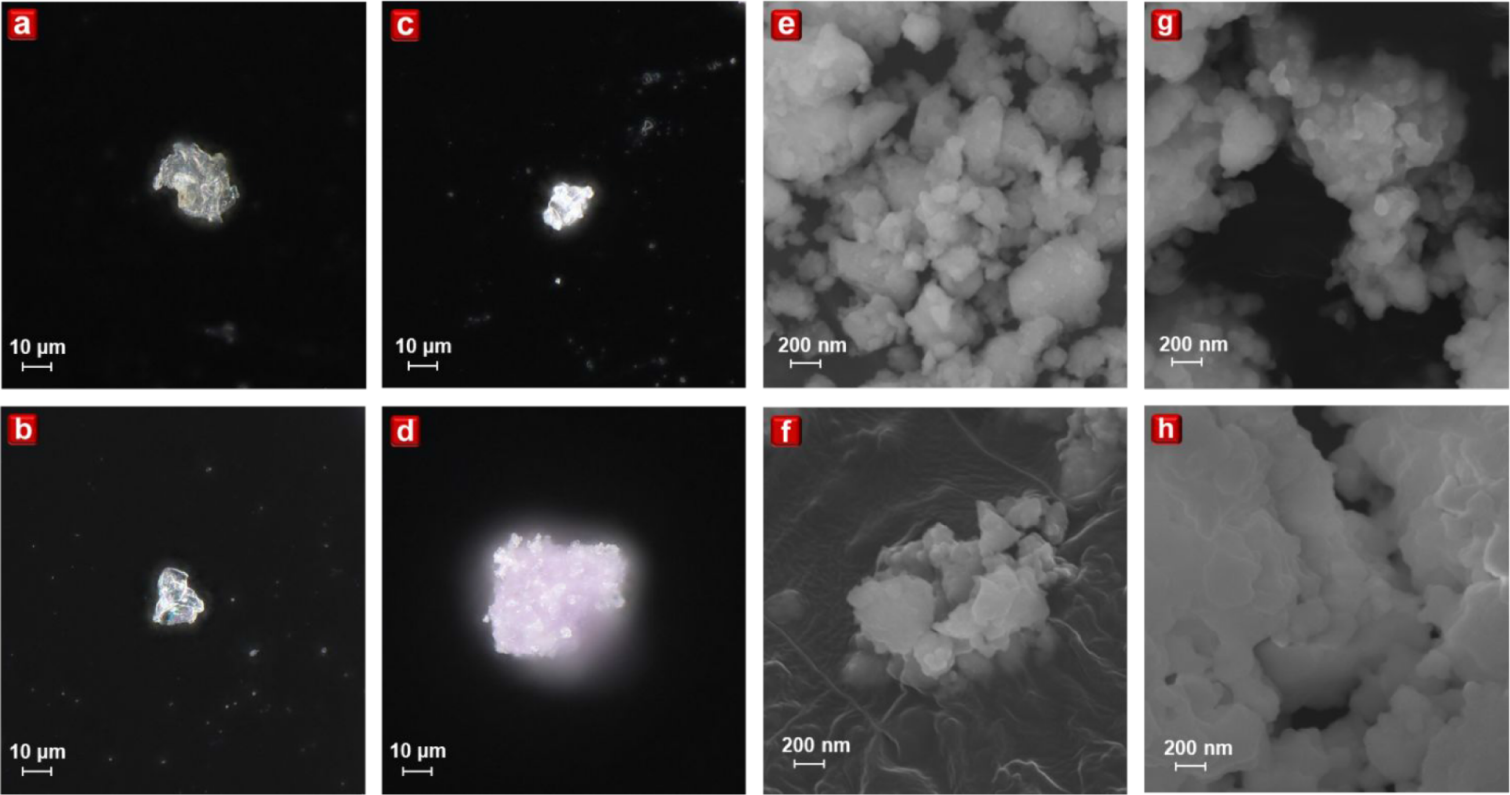
OM images of Cs_2_AgIn_1–*x*_Cr_*x*_Cl_6_ with *x* = (a) 0.00, (b) 0.01, (c) 0.05, and (d) 0.10 captured under 1500× magnification. SEM images of Cs_2_AgIn_1–*x*_Cr_*x*_Cl_6_ with *x* = (e) 0.00, (f) 0.01, (g) 0.05, and (h) 0.10 captured under 100 000× magnification.

To obtain in-depth morphological insights, scanning electron microscopy (SEM) was employed to capture images of Cs_2_AgIn_1–*x*_Cr_*x*_Cl_6_ (*x* = 0.00, 0.01, 0.05, and 0.10) at magnifications of 100 000× (Figure 2e–h) and 20 000× (Figure S4a–d). At higher magnification, the particle sizes of the *x* = 0.00, 0.01, and 0.05 samples were in the range of a few hundred nanometers, indicating their potential applicability for mini-LED technology with appropriate sieving. Despite the tendency to aggregate, the *x* = 0.10 sample showed particle sizes comparable to or slightly larger than those of the *x* = 0.00, 0.01, and 0.05 samples. This suggests that the broadening of the Raman peak observed in the *x* = 0.10 is not attributed to the particle size reduction but rather to the introduction of the [CrCl_6_] mode or structural distortions. While SEM allows for higher magnification, it does not provide information on the transparency and body color of the materials. Therefore, OM and SEM are complementary techniques, offering a comprehensive overview of morphological properties.

### Photoluminescence

To determine the optical properties of the Cs_2_AgIn_1–*x*_Cr_*x*_Cl_6_, the photoluminescence excitation (PLE) spectra and the corresponding diffuse reflectance spectra (DRS) of Cs_2_AgIn_1–*x*_Cr_*x*_Cl_6_ were analyzed, as shown in Figure 3a,b, respectively. All spectra of Cr^3+^ doped samples consist of three prominent excitation bands peaking around 380, 585, and 825 nm. The latter two are typical bands for Cr^3+^ in 6-fold octahedral coordination. The band at 585 nm corresponds to the ^4^ *A*_2_ → ^4^ *T*_1_ transition, while the band at 825 nm corresponds to the ^4^ *A*_2_ → ^4^ *T*_2_ transition. The peak in the PLE spectra at 380 nm corresponds well to the absorption edge observed in the DRS for doped samples. The noticeable difference between the absorption edge location of doped and undoped samples (marked by the dashed line in Figure 3b) suggests that the 380 nm band is associated with the Cr^3+^ dopant (^4^ *A*_2_ → ^4^ *T*_1_(*P*)) rather than host absorption, contradicting previous assertions in the literature.^26^ Additionally, as photoelectric measurements will later confirm, there is a charge transfer transition (CTT) band of Cr^3+^ at 355 nm. Notably, as the concentration of Cr^3+^ increases, there is a slight wavelength shift in the spectra maxima from 580 and 820 nm in the *x* = 0.01 sample to 585 and 825 nm in the *x* = 0.10 sample. The photoluminescence (PL) spectra in Figure 3c feature the broadband spin-allowed ^4^ *T*_2_ → ^4^ *A*_2_ transition, with the peak slightly shifting from 1000 to 1005 nm as Cr^3+^ concentration increases. The PL intensity increases by 2.3 when the Cr^3+^ concentration rises from *x* = 0.01 to 0.05 but remains constant for *x* = 0.10 sample. Relative quantum efficiency (QE) was estimated by considering the changes in absorption presented in Figure 3b. By evaluating the absorption values of *x* = 0.01, 0.05, and 0.10 samples at the peak of the ^4^ *T*_1_ → ^4^ *A*_2_ band (565 nm) and subtracting the baseline absorption of the pristine sample, the absorption of *x* = 0.05 and *x* = 0.10 samples increases by factors of 2.8 and 4.1, respectively, relative to the *x* = 0.01 sample (Table S3). However, since the PL intensity increased by only 2.3, concentration quenching effects limit the relative QE of *x* = 0.05 and 0.10 samples to 84% and 57% of the QE value for x = 0.01 sample, respectively. Nevertheless, the actual QE values of all samples are likely lower due to thermally induced quenching, as evidenced by the increase in intensity when the temperature drops below room temperature, which will be discussed in detail in subsequent sections.

**Figure 3:**
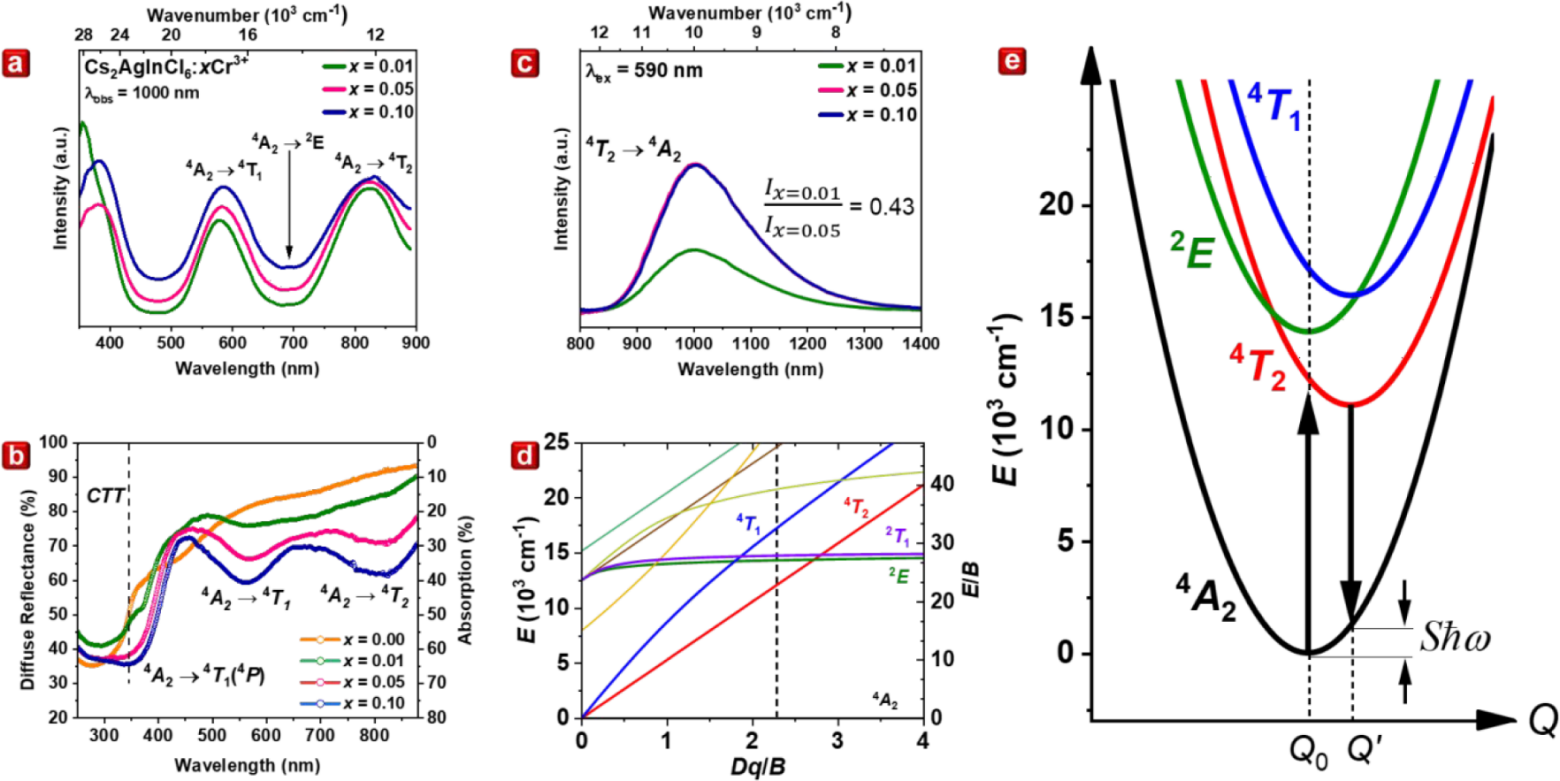
(a) RT PLE spectra observed at 1000 nm of Cs_2_AgIn_1–*x*_Cr_*x*_Cl_6_ with *x* = 0.01, 0.05, and 0.10. (b) Diffuse reflectance spectra of Cs_2_AgIn_1–*x*_Cr_*x*_Cl_6_ with *x* = 0.00, 0.01, 0.05, and 0.10. (c) PL spectra of Cs_2_AgInCl_6_:*x*%Cr^3+^ upon 590 nm excitation of Cs_2_AgIn_1–*x*_Cr_*x*_Cl_6_ with *x* = 0.01, 0.05, and 0.10. (d) Tanabe-Sugano diagram. (e) Configurational coordinate diagram representing the low field Cr^3+^ system.

Considering the observed energies of the ^4^ *A*_2_ → ^4^ *T*_2_ and ^4^ *A*_2_ → ^4^ *T*_1_ taken from the PLE spectra, it is possible to obtain the values of the crystal field strength parameter *Dq* and the Racah parameter *B*, according to the equations:^41^

1 10Dq=E(A24→T24)

2 $$B=\frac{\frac{x^{2}}{Dq}-10x}{15\left(\frac{x}{Dq}-8\right)}$$

3 E(A24→E4)B=3.05CB+7.9−1.8BDq

Here, the *E*(^4^ *A*_2_ *→* ^4^ *T*_2_) is the peak energy of the ^4^ *A*_2_ →^4^ *T*_2_ PLE band, while *x* is the energy difference between the maxima of ^4^ *T*_1_ and ^4^ *T*_2_ bands in the PLE spectrum: *x* = *E*(^4^ *A*_2_ → ^4^ *T*_1_) – *E*(^4^ *A*_2_ → ^4^ *T*_2_). The energy of the^2^ *E* was obtained assuming that a small feature in the PLE spectra located at 700 nm corresponds to the *E*(^4^ *A*_2_ → ^2^ *E*) transition. Determining this transition energy is essential for calculating the Racah parameter *C*. To calculate the crystal field splitting parameter *Dq* and the Racah parameter *B*, we assumed that the energies of the ^4^ *T*_2_ and ^4^ *T*_1_ states correspond to the peak positions of their respective PLE bands. This approach is consistent with the methodology discussed in the literature.^42^ The resulting values of crystal field parameters are presented in Table S4.

Based on the parameters presented in Table S4, the Tanabe–Sugano (T–S) diagram of the system was constructed in Figure 3d, which showcases the relative positions of the ground state ^4^ *A*_2_ and the first four excited states ^2^ *E*, ^2^ *T*_1_, ^4^ *T*_2_, and ^4^ *T*_1_ of Cr^3+^ ions in the Cs_2_AgInCl_6_ host. The *x*-axis of the T–S diagram is expressed by the crystal field splitting parameter *Dq*, while the *y*-axis is in terms of the energy *E*. Both are scaled by the Racah parameter *B*. The obtained diagram indicates that the ^4^ *T*_2_ state is the lowest excited state, which is consistent with the observed broadband emission and confirms that Cr^3+^ ions occupy octahedral sites under a weak crystal field. Since the T–S diagram does not account for the lattice relaxation, a discrepancy occurs between the ^4^ *T*_2_ position in the diagram and the actual peak of the broadband ^4^ *T*_2_ → ^4^ *A*_2_ emission. The lattice relaxation can be described by the quantity *S*ℏω, where ℏω is an effective phonon frequency, and *S* is the Huang–Rhys parameter.^41^ Knowing the Stokes shift between absorption ^4^ *A*_2_ → ^4^ *T*_2_ and emission ^4^ *T*_2_ → ^4^ *A*_2_, the electron–lattice coupling can be determined as half of the Stokes shift with energy 2*S*ℏω ≈ 2400 cm^–1^. This information was used to construct a configuration coordinate diagram (Figure 3e), where each electronic state is represented by a parabola, illustrating the harmonic potential responsible for lattice vibrations.

### Temperature-Dependent Analysis

The temperature-dependent PL spectra of Cs_2_AgIn_1–*x*_Cr_*x*_Cl_6_ (*x* = 0.01, 0.05, and 0.10) are presented in Figure 4a–c. As the temperature increases, all samples exhibit a noticeable shift toward longer wavelengths (i.e., from 960 to 1000 nm), accompanied by an expansion in *fwhm* from 120 to 190 nm. Figure 4d shows temperature dependence of total (integrated) emission intensity, as shown in Figure 4a–c. The *x* = 0.01 sample displays a steady increase in intensity up to 225 K, followed by a sharp decline, likely due to temperature-induced quenching. Similar trends of nonradiative quenching are observed just below room temperature in the higher concentration samples, *x* = 0.05 and *x* = 0.10. The *T*_50_ was calculated to be around 300 K for each sample exhibiting high thermal quenching. Furthermore, the initial increase in intensity can be attributed to the increase in the absorption coefficient associated with an increasing phonon population.^43^ However, in samples with higher Cr^3+^ concentrations, this initial rise is suppressed, and an opposite trend is observed in the cryogenic region, where emission intensity decreases with temperature. This concentration-dependent effect is likely due to thermally assisted energy transfer between Cr^3+^ ions, ultimately leading to quenching by luminescence deactivation centers. Due to the significant Stokes shift, the spectral overlap between the absorption and emission spectra strongly depends on temperature broadening, which increases the energy transfer probability and results in the observed temperature dependence.

**Figure 4:**
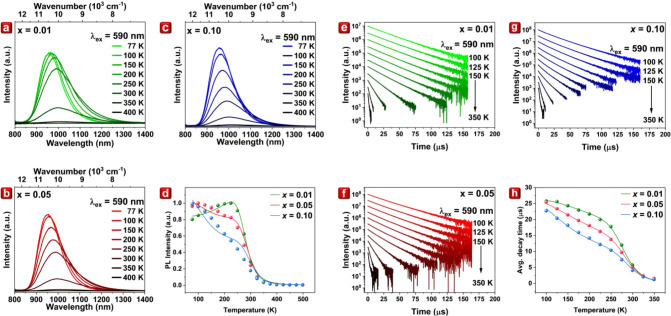
Temperature-dependent PL spectra of Cs_2_AgIn_1–*x*_Cr_*x*_Cl_6_ with *x* = (a) 0.01, (b) 0.05, and (c) 0.10. (d) Total (integrated) PL intensities of Cs_2_AgIn_1–*x*_Cr_*x*_Cl_6_ with *x* = 0.01, 0.05, and 0.10. (e) Temperature-dependent decay profiles of Cs_2_AgIn_1–*x*_Cr_*x*_Cl_6_ with *x* = (e) 0.01, (f) 0.05, and (g) 0.10. (h) Calculated average decay times of Cs_2_AgIn_1–*x*_Cr_*x*_Cl_6_ with *x* = 0.01, 0.05, and 0.10.

To further comprehend the kinetics of the radiative and nonradiative processes, the temperature-dependent decay curves of Cs_2_AgIn_1–*x*_Cr_*x*_Cl_6_ (*x* = 0.01, 0.05, and 0.10) at 590 nm excitation in 100–350 K range were measured and presented in Figure 4e–g. As the profiles exhibit deviations from single-exponentiality, average decay times were computed using the following formula (eq 4):

4 $$\tau_{av}=\frac{\int I\left(t\right)tdt}{\int I\left(t\right)dt}$$

where *I­(t)* is the emission intensity at time *t*, the calculated average decay times are demonstrated in Figure 4h. At 100 K, the decay times for the Cs_2_AgIn_1–*x*_Cr_*x*_Cl_6_ (*x* = 0.01, 0.05, and 0.10) samples are 25.8 μs, 25.45 μs, and 22.7 μs, respectively. As the temperature increases, the decay times decrease at different rates for each sample. For the *x* = 0.01 sample, there are two distinct phases of decay time reduction: an initial gradual decline up to 250 K, followed by a steeper decrease above this threshold. The initial decline of the *x* = 0.01 sample can be attributed to the enhanced thermal population of odd-parity vibrational modes, contributing to the increased radiative probability and PL intensity. For the *x* = 0.05 and 0.10, the temperature-dependent luminescence decay time is qualitatively similar; however, the onset of the initial decay time reduction occurs earlier, leading to a steeper decline. This behavior is associated with the thermally assisted concentration quenching observed in the PL intensity. The complex interplay between PL intensity and decay time temperature variations requires a comprehensive model that accounts for all relevant factors: the temperature-dependent increase in radiative transition probabilities, thermal quenching, and thermally assisted energy transfer.

The thermally enhanced radiative transition rate can be understood by assuming that activation of odd-parity vibrations of the Cr^3+^–Cl_6_^6–^ manifold relaxes the parity selection rule for the *d*-*d* forbidden transitions. This affects both the absorption cross-section *σ*(*T*) from the ground state as well as radiative decay probability*τ* ^–1^(*T*) of the excited state:^44^

5 $$\sigma \left(T\right)=\sigma_{0}\cot h\left(\frac{\hbar \omega }{2kT}\right)$$

6 $$\tau^{-1}\left(T\right)=\tau_{0}^{-1}\cot h\left(\frac{\hbar \omega }{2kT}\right)$$

where *σ*_0_ and $\tau_{0}^{-1}$ are the 0 K values of absorption cross-section and radiative probability, respectively. Theℏω corresponds to the effective energy of odd-parity vibrations coupling to the electronic transitions.

The effect of thermally assisted energy transfer can be modeled as a nonradiative process with activation energy on the order of the *S*ℏω and probability inversely dependent on the distance between the Cr^3+^ ions, which decreases with concentration. The “activation energy” is related to increasing spectral overlap between the absorption and emission band of Cr^3+^ centers, enabling the energy transfer process.

The relevant formula describing PL intensity dependence and decay times with respect to temperature, taking into account the temperature dependence of radiative rate—equations (eq 5) and (eq 6), nonradiative quenching and energy transfer are as follows:

7 $$I\left(T\right)=\frac{A\cot h\left(\frac{\hbar \omega }{2kT}\right)}{1+\tau_{0}\tan h\left(\frac{\hbar \omega }{2kT}\right)\cdot \left[s\exp\left(-\frac{E_{nr}}{kT}\right)+p\exp\left(-\frac{E_{et}}{kT}\right)\right]}$$

8 $$\tau^{-1}\left(T\right)=\tau_{0}^{-1}\cot h\left(\frac{\hbar \omega }{2kT}\right)+s\exp\left(-\frac{E_{nr}}{kT}\right)+p\exp\left(-\frac{E_{et}}{kT}\right)$$

Here, *A* is a constant dependent on the absorption cross-section σ_0_; *s* and *E*_*nr*_ represent the frequency factor and activation energy of the nonradiative quenching; *E*_*et*_ and *p* represent the activation energy and probability of the thermally activated energy transfer process. The PL intensity and decay time fitting results are presented as solid lines in Figure 4d,h, respectively. The parameters obtained from the fitting are showcased in Table S5. In the fitting procedure*τ*_0_ , ℏω, *E*_*nr*_, *E*_*et*_ , *s* were constrained to have the same value across all data sets, while *p* (which explicitly depends on Cr^3+^–Cr^3+^ distance) was set as a free parameter, along with *A*. The fitting curves for luminescence decay times fit the experimental results very closely, while the fitting curves for the PL intensity data show some deviation from the experimental results. However, the model correctly accounts for the initial intensity rise for low Cr^3+^ concentration.

The effective odd-parity phonon energy ℏω obtained from the experiment (430 cm^–1^) is consistent with expectations, as is the activation energy and frequency factor for the nonradiative quenching: 3030 cm^–1^ and 2 × 10^12^ s^–1^, respectively. The activation energy for energy transfer is on the order of phonon energy (∼250 cm^–1^), which aligns well with the model’s assumption. The probability factor *p* for energy transfer is 8 orders of magnitude lower than frequency factor *s* but follows the predicted increase in value with the increase in concentration. It should be noted that the obtained value for *x* = 0.01 sample from PL intensity dependence is effectively zero, as it is less than zero and falls below the standard error threshold.

Considering the configuration diagram, we can exclude the occurrence of nonradiative relaxation resulting from the crossing of energy curves between the excited ^4^ *T*_2_ and ground state ^4^ *A*_2_ (so-called intersystem relaxation). The activation energies obtained from fitting the intensity and time–temperature dependence are significantly lower than those associated with crossing ^4^ *T*_2_ and ^4^ *A*_2_ levels, which are notably higher (∼15 000 cm^–1^). Another possible mechanism for the quenching of luminescence intensity is autoionization, where an electron in an excited state may be thermally transferred to the conduction band (CB) due to phonon interaction, thereby contributing to the generation of current carriers within the band.

### Photoelectric Measurements and Quenching Model

The analysis presented in the previous section proves that the luminescence quenching mechanism cannot be linked to intersystem crossing within the Cr^3+^ energy structure. The structural characterization does not indicate that sample quality influences thermal quenching, especially since the thermal quenching of chloride double perovskites has been widely reported in the literature. Therefore, thermal quenching is most plausibly an intrinsic property of the energy structure of chloride double perovskites. A detailed understanding of the energetic structure of the phosphor as a whole can be gained by studying its photoelectric properties (photoconductivity excitation spectra), which reveal the location of localized dopant states and the band states of the host material. First, photocurrent excitation spectra may indicate the occurrence of the autoionization process, where the excited dopant ion transfers electrons to the CB or holes into the valence band.^23^ Second, such studies can reveal the location of the charge transfer state, therefore elucidating the location of the dopant within the bandgap of the material.

Figure 5a displays the photocurrent excitation (PCE) spectra (dashed line) for Cs_2_AgIn_1–*x*_Cr_*x*_Cl_6_ with *x* ranging from 0 to 0.05. For *x* = 0, the PCE spectra reveal a broad band between 250–350 nm, peaking at 310 nm, which is attributed to the free excitonic absorption. In the Cr^3+^-doped sample, an additional band appears at 355 nm in the PCE spectra, which is related to a charge transfer transition (CTT); the electron from the ground Cr^3+^ state is transferred to the CB. It should be noted that excitation in the visible range related to the transition from ^4^ *A*_2_ to the ^4^ *T*_2_ or ^4^ *T*_1_ of Cr^3+^ ions states does not result in the observation of photocurrent. This allows us to exclude the hole-type thermal quenching process, as observed in the case of Cr^3+^ activated oxide materials.^23^ The kinetics of the process occurring in the tested systems are presented in Figure 5b. The material with *x* = 0.05 was chosen as an example, although the described process applies to all Cr^3+^ ion-activated materials tested. Specific energy levels of the Cr^3+^ ion are marked within the bandgap. The position of the Cr^3+^ ground state was determined based on the ionization transition CTT observed in the photocurrent excitation spectra. The diagram clearly shows that luminescence quenching is unrelated to the autoionization process, as the ^4^ *T*_2_ state is significantly lower than the CB, making the ionization transition energy exceed 15 000 cm^–1^ in Cs_2_AgIn_1–x_Cr_*x*_Cl_6_. Since this quenching is not associated with either the crossing point of the excited ^4^ *T*_2_ and ground state ^4^ *A*_2_ of Cr^3+^ or autoionization, additional levels involved in the quenching process (marked in red) must be considered. The processes with an activation energy of 3030 cm^–1^ indicate strong quenching of the Cr^3+^ luminescence, attributable to transitions from the ^4^ *T*_2_ emission state to a state lying deep in the energy bandgap. This state is likely related to the self-trapped exciton (STE), which often occurs in this type of material.^45^ This situation is better illustrated in the configuration diagram in Figure 5c, which elucidates the significance of the strong lattice relaxation of the STE state for the quenching process. As the temperature increases, electrons are activated from the ^4^ *T*_2_ state to the STE state with the activation energy *E*_*nr*_, leading to nonradiative deactivation in this material. We propose that this is a general mechanism underlying the abnormal thermal quenching of Cr^3+^ luminescence in chloride double perovskite compounds where the excitonic state STE occurs, as supported by previously mentioned literature examples. Because of the large Stokes shift of the STE, the interference of the STE states with the ^4^ *T*_2_ emitting state appears unavoidable, compromising the potential of chloride double perovskites as Cr^3+^-activated NIR phosphors.

**Figure 5:**
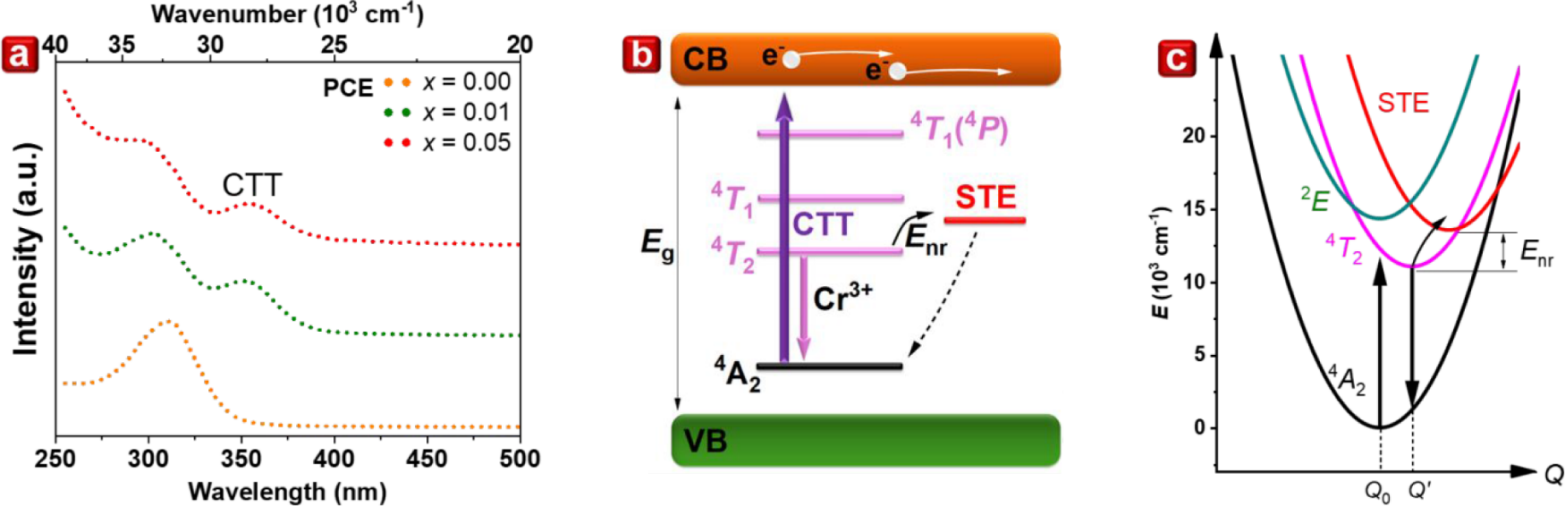
(a) PCE spectra of Cs_2_AgIn_1–*x*_Cr_*x*_Cl_6_, *x* = 0–0.05. (b) Energy level diagram describing the kinetics of the observed processes. (c) Configurational coordinate diagram representing the STE and *E*_nr_.

## Conclusions

In conclusion, a series of Cs_2_AgInCl_6_ doped with different concentrations of Cr^3+^ is achieved using a ball-milling mechanochemistry method. This approach allowed precise chemical composition control without the need for further sintering. The crystal structures of Cs_2_AgIn_1–*x*_Cr_*x*_Cl_6_ (*x* = 0.00, 0.01, 0.05, and 0.10) were thoroughly analyzed using Raman spectra and Rietveld refinement using synchrotron XRD, confirming the successful Cr doping. High magnification OM revealed the transparent crystal morphology and the color change from white to purple as the Cr concentration increased. Additionally, the SEM showed that the particle sizes of the materials were around a few hundred nanometers. PLE and DRS were employed to explore the optical properties of the undoped host materials and the Cr-doped Cs_2_AgIn_1–*x*_Cr_*x*_Cl_6_ phosphors, distinguishing between host absorption, charge transfer transitions, and activator absorption. The PL spectra of Cs_2_AgIn_1–*x*_Cr_*x*_Cl_6_ cover 850–1350 nm, providing a broadband emission that fills the gap between the NIR and SWIR regions. The Tanabe–Sugano diagram and configurational coordinate diagram illustrate the electronic transition processes. Temperature-dependent PL and decay curve revealed the unexpected changes in lifetime and luminescent intensity at different temperatures, which were explained by considering the increase in the absorption coefficient, thermal quenching, and thermal-activated energy transfer. The luminescent intensity and lifetime changes also fit well with the developed formula that accounts for the temperature-dependent increase in radiative transition probabilities, thermal quenching, and thermally assisted energy transfer. Moreover, PCE spectra were compared with the absorption spectra to elucidate the quenching mechanism. The results indicate that quenching is not due to thermal ionization or electronic crossing between the ^4^ *T*_2_ and ^4^ *A*_2_ levels but rather from the electronic transition from the ^4^ *T*_2_ state to the STE state, which is deactivated nonradiatively in Cs_2_AgIn_1–*x*_Cr_*x*_Cl_6_.The significant Stokes shift of the self-trapped exciton (STE) leads to unavoidable interference between the STE states and the Cr^3+ 4^ *T*_2_ emitting state. We propose that this is a general mechanism underlying the abnormal thermal quenching of Cr^3+^ luminescence in halide double perovskite compounds. This finding suggests that halide double perovskites, where the self-trapped exciton (STE) occurs, may not be the most suitable hosts for Cr^3+^ -activated luminescent materials. It also indicates the potential need to explore alternative strategies for developing halide double perovskite-based NIR phosphors.

## Method

### Materials

All the chemicals were used as received without further purification. Cesium chloride (CsCl, ≥99%) and silver chloride (AgCl, 99.9%) were purchased from Thermo Fisher Scientific. Indium­(III) chloride (InCl_3_, 99.99%) was purchased from Nova-Matls. Chromium­(III) chloride hexahydrate (CrCl_3_·6H_2_O, 96%) was purchased from Sigma-Aldrich.

### Synthesis

The synthesis of Cs_2_AgIn_1–*x*_Cr_*x*_Cl_6_ powder was carried out through a ball milling process, utilizing stoichiometrically measured precursors, including CsCl, AgCl, InCl_3_, and CrCl_3_·6H_2_O. The precursor mixture was transferred into a ZrO_2_ jar with ZrO_2_ balls, followed by mechanical milling. The milling procedure was done in several cycles. Each cycle consists of 15 min milling at 30 Hz and 5 min resting at 3 Hz. The total ball milling duration was 2–10 h to obtain Cs_2_AgIn_1–*x*_Cr_*x*_Cl_6_ powder. After the ball milling reaction, the powder was briefly sieved through a strainer to separate the powder and the ZrO_2_ balls. Finally, the Cs_2_AgIn_1–*x*_Cr_*x*_Cl_6_ powder could be obtained. Detailed characterizations of the synthesized powder are listed in the Supporting Information.

## Supplementary Material



## Notes

Characterization; structural information; Rietveld refinement; SEM; OM; absorption; photoluminescence parameters (PDF)
