# Supplementary material for: Understanding the Limitations of Chloride Double Perovskites as Hosts for Stable Cr3+ Luminescence
Source: Inorg Chem. 2025 Jul 16;64(29):15184–92. doi: 10.1021/acs.inorgchem.5c02293 (PMC12308787; doi:10.1021/acs.inorgchem.5c02293)
Supplement: Supplementary file 1 [file ic5c02293_si_001.pdf]

Supporting Information

Understanding the Limitations of Chloride Double Perovskites as Hosts for Stable Cr^{3+} Luminescence

Kuan-Yi Lee,[Ω†] Hsiu-Kai Yang,[Ω†] Tadeusz Leśniewski,[Ω§Φ] Mikołaj Kamiński,[§] Natalia Majewska,[‡,§] Jakub Gnyp,[§] Wei-Lun Su,[†] Yi-Ting Tsai,[†] Sebastian Mahlik*[§] and Mu-Huai Fang*[†]

[†]Research Center for Applied Sciences, Academia Sinica, Taipei 11529, Taiwan.

[§]Institute of Experimental Physics, Faculty of Mathematics, Physics and Informatics, University of Gdansk, Wita Stwosza 57, 80-308 Gdansk, Poland.

[Φ]Faculty of Physics, Kazimierz Wielki University in Bydgoszcz, Powstańców Wielkopolskich 2, 85-090, Bydgoszcz, Poland

[‡]Faculty of Chemistry, Adam Mickiewicz University, Uniwersytetu Poznańskiego 8, 61-614 Poznań, Poland.

Email: sebastian.mahlik@ug.edu.pl (S.M.) and fangmuhuai@gate.sinica.edu.tw (M.H.F)

Characterization:

The high-resolution synchrotron XRD patterns were characterized at the National Synchrotron Radiation Research Center (NSRRC, Taiwan) under the TPS-19A1 beamline with a wavelength of 0.61992 Å. The scanning electron microscope (SEM) ThermoFisher Phenom Pharos collects images with magnification of 20 000× and 100 000×. Keyence VHX-7000 characterizes the optical microscope images with magnification of 150× and 1500×. Raman spectra were recorded using a confocal micro-Raman system equipped with a microscope and a Horiba Jobin Yvon LabRAM Aramis spectrometer with a laser providing excitation light at 532 nm with the 1200 l/mm grating. The PLE spectra were obtained using a specialized arrangement compromising of EQ99X laser-driven Xe light source (Energetiq) coupled to a self-made grating monochromator operating between 350-1000 nm as the excitation source and the Andor SR-500i-D1 spectrometer equipped with a CCD camera (DU490A-1.7) operating in the 600-1700 nm wavelength as luminescence detector. The Diffuse Reflectance Spectra was measured using a Quantaurus-QY Absolute PL quantum yield spectrometer (Hamamatsu). The temperature-dependent photoluminescence spectra were recorded using an Andor SR-500i-D1 spectrometer with a CCD camera

(DU490A-1.7) operating in the 600–1700 nm wavelength range. The excitation source employed was identical to that used in the PLE setup. The temperature control was achieved through the THMS600 temperature-controlled stage with the LNP95 LN_2-vapour cooling pump (Linkam Scientific), allowing measurement in the 77–600 K temperature range. The decay profiles in the NIR range were acquired employing a custom-made setup comprising of SIGLANT SDG2082X function generator of square-shaped light pulses with automatically adjusted repetition, a 590 nm LED as excitation source, and a RIGOL HDO4204 200 MHz Digital Oscilloscope, and APD110C/M Avalanche Photodetector operating in the spectral range 900–1700 nm. The power of the light source of pc-LED and halogen lamp was determined by Thorlab PM100D. Photocurrent excitation (PCE) spectra were recorded using a custom-built setup. This system included a 150 W xenon lamp (LOT Quantum Design) connected to a grating monochromator (Omni-λ 1509), which provided excitation light in the 250–1000 nm range. A digital electrometer (Keysight B2987A) measured the photocurrent. To improve the signal-to-noise ratio, the excitation light was modulated at 5 Hz with an optical chopper, and the photocurrent signal was detected using a lock-in amplifier (Signal Recovery 7270, Ametek Scientific Instruments).

Energy structure calculation:

Presented electron structure was computed with DFT in Quantum Espresso 7.3[1,2] using GBRV high-throughput USPP pseudopotentials.[3] The calculations employed GBRV pseudopotentials within the framework of Quantum ESPRESSO, utilizing the Perdew–Burke–Ernzerhof (PBE) exchange-correlation functional and ultrasoft pseudopotentials. A cubic structure of 40 atoms was evaluated for the self-consistent field (SCF) calculations. Atomic positions and dimensions of the structure were retrieved from the Materials Project for $Cs_2InAgCl_6$ (mp-1096926) from database version v2025.02.12.post. An energy cutoff of 60 Ry was selected, alongside Marzari-Vanderbilt-DeVita-Payne cold smearing with the Gaussian spreading of 0.02 Ry for Brillouin-zone integration, convergence threshold 1.0d-9, the default value 0.7 for the mixing beta and the Monkhorst-Pack k-grid 10×10×10 with offset 1×1×1. For the bands calculations themselves energy cutoff was set to 60 Ry and 240 Ry for the wavefunctions and charge density, respectively. 220 Kohn-Sham states were computed, roughly ranging from −86 eV to 11 eV. Self-consistent field (SCF) and non-self-consistent field (NSCF) calculations for band structure analysis indicated that the system exhibits a point group symmetry of O_h ($m\bar{3}m$). All high-symmetry points were provided with weights 30, with the sole exception of the last point, resulting in 271

energy values for consecutive k-points for each band. Due to erroneous calculations of the k-path cumulative length by Quantum Espresso, the corrected lengths were computed in Python 3.13 with the NumPy library. An energy threshold of −6.7969 eV was applied for better readability, as shown in Figure S5.

Additionally, projected density of states (PDOS) of atoms in the conventional cell was computed using the tetrahedron method on a 40x40x40 Monkhorst-Pack k-grid with 1x1x1 offset. Other parameters, such as the Gaussian spreading, convergence threshold, mixing beta and energy cutoffs were applied as in the case of bands calculations. Resulting PDOS were stacked per element in the conventional cell. As density of states clearly shows, computed direct band gap is smaller than the one observed experimentally due to a forbidden transition – the actual transition takes place between states of higher density.

Computations were carried out using the computers of the Centre of Informatics Tricity Academic Supercomputer & Network. SCF calculations were parallelized on 4 nodes with 48 processors each and achieved convergence after 14 iterations in 3m 4.16s, returning the Fermi energy 4.2969 eV and the total energy −3024.03983839 Ry, with estimated SCF accuracy of 1.4E−10 Ry. Non-SCF calculations for bands were also performed on 4 nodes with 48 processors each, taking 31m 27.41s. The NSCF calculation for DOS was parallelized on 10 nodes with 48 processors each and took 1h 25m to complete. Projected DOS calculations were performed on a single computational node with 48 processors and took 15m 30.62s to be finished.

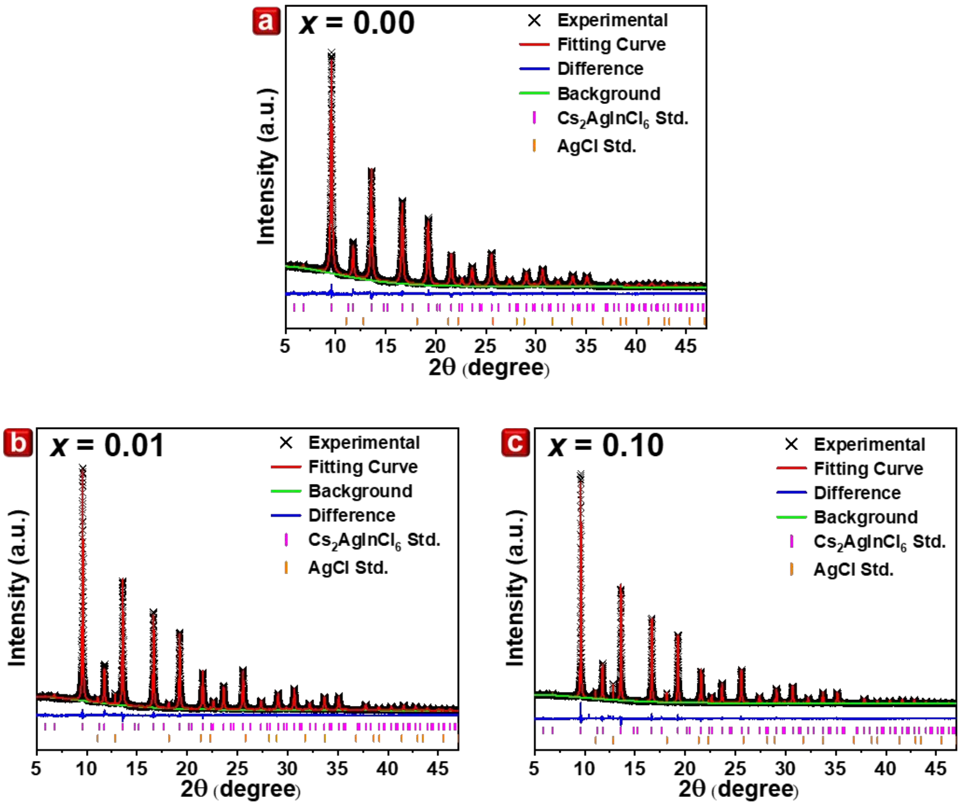

Figure S1. Rietveld refinement of $Cs_2AgIn_{1-x}Cr_xCl_6$ with x = (a) 0.00, (b) 0.01, and (c) 0.10.

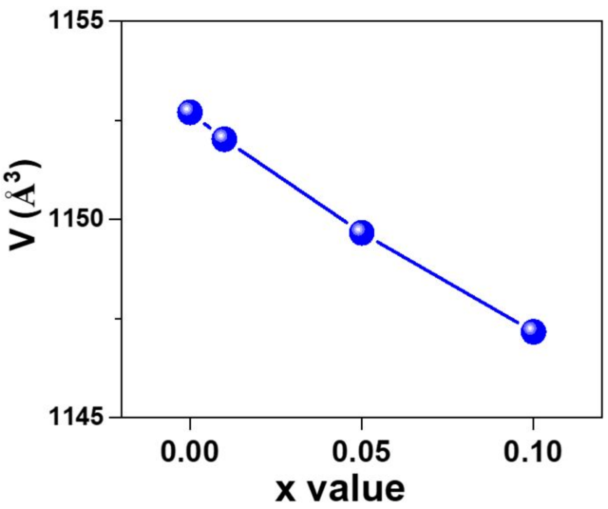

Figure S2. Unit cell volume of $Cs_2AgIn_{1-x}Cr_xCl_6$ with x = 0.00, 0.01, 0.05, and 0.10.

Table S1. Refined atomic positions, atomic displacement parameters, and occupancy values of $Cs_2AgIn_{1-x}Cr_xCl_6$ with x = 0.00, 0.01, 0.05, and 0.10.

***x* = 0.00**

Site	Np	*x*	*y*	*z*	*Occ*	*Beq*
Cs1	8	0.25	0.25	0.25	1	1.96(1)
Ag1	4	0.5	0.5	0.5	1	2.89(4)
In1	4	0	0	0	1	0.60(2)
Cl1	24	0.2418(3)	0	0	1	2.28(2)

***x* = 0.01**

Site	Np	*x*	*y*	*z*	*Occ*	*Beq*
Cs1	8	0.25	0.25	0.25	1	1.99(1)
Ag1	4	0.5	0.5	0.5	1	2.90(3)
In1	4	0	0	0	1	0.77(2)
Cl1	24	0.2431(2)	0	0	1	2.34(2)

***x* = 0.05**

Site	Np	*x*	*y*	*z*	*Occ*	*Beq*
Cs1	8	0.25	0.25	0.25	1	2.07(1)
Ag1	4	0.5	0.5	0.5	1	2.42(3)
In1	4	0	0	0	0.886(4)	0.533(2)
Cr1	4	0	0	0	0.114(4)	0.533(2)
Cl1	24	0.2415(2)	0	0	1	2.29(2)

***x* = 0.10**

Site	Np	*x*	*y*	*z*	*Occ*	*Beq*
Cs1	8	0.25	0.25	0.25	1	2.10(1)
Ag1	4	0.5	0.5	0.5	1	2.36(3)
In1	4	0	0	0	0.852(4)	0.65(2)
Cr1	4	0	0	0	0.148(4)	0.65(2)
Cl1	24	0.2405(2)	0	0	1	2.31(2)

Table S2. Refined parameters of $Cs_2AgIn_{1-x}Cr_xCl_6$ with x = 0.00, 0.01, 0.05, and 0.10.

***x* =**	**0.00**	**0.01**	**0.05**	**0.10**
$Cs_2AgInCl_6$ (wt%)	99.17(8)	98.38(2)	98.11(3)	98.55(1)
AgCl (wt%)	-	1.62(2)	1.89(3)	1.45(1)
a (Å)	10.4851(1)	10.48302(5)	10.47585(4)	10.46826(4)
V (Å^3)	1152.70(3)	1152.02(2)	1149.66(1)	1147.16(1)
R_{wp} (%)	2.76	3.02	2.95	3.17
R_p (%)	2.17	2.38	2.32	2.41
GOF	1.19	1.08	1.04	1.51

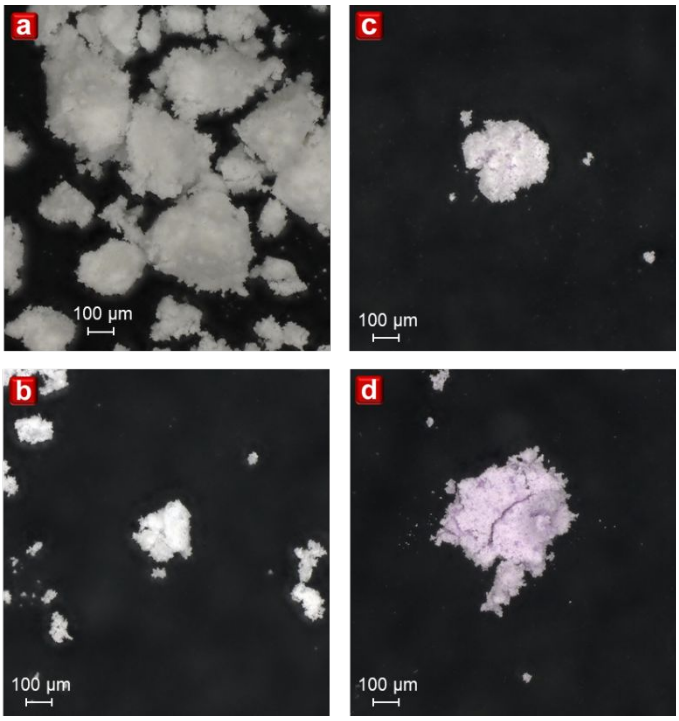

Figure S3. OM images of $Cs_2AgIn_{1-x}Cr_xCl_6$ with x = (a) 0.00, (b) 0.01, (c) 0.05, and (d) 0.10 captured under 150× magnification.

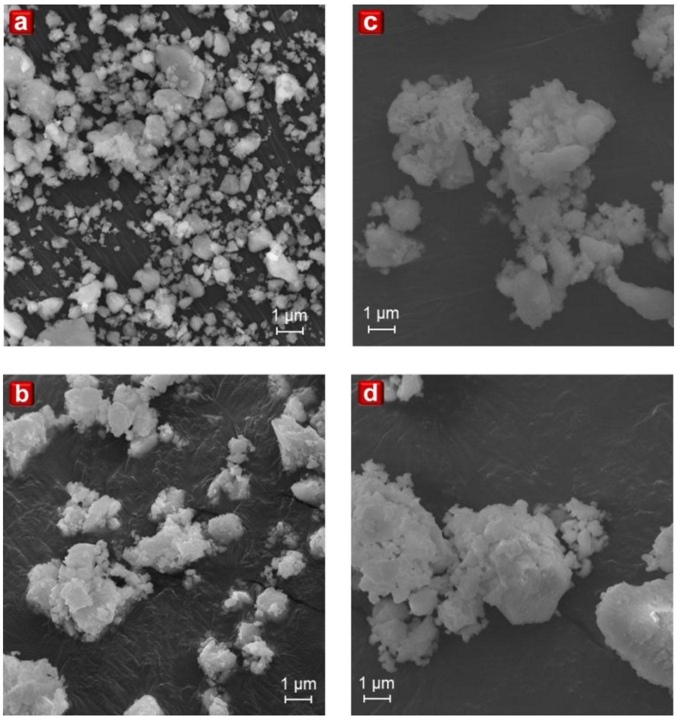

Figure S4. SEM images of $Cs_2AgIn_{1-x}Cr_xCl_6$ with x = (a) 0.00, (b) 0.01, (c) 0.05, and (d) 0.10 captured under 20 000× magnification.

Table S3. Relative absorption and QE of $Cs_2AgIn_{1-x}Cr_xCl_6$ with x = 0.00, 0.01, 0.05, and 0.10.

	Total absorption at 565 nm	Cr^{3+} absorption at 565 nm (baseline subtracted)	Relative Cr^{3+} absorption at 565 nm *	Relative PL intensity *	Relative quantum efficiency *
***x* = 0.00**	0.19	0	0	0	-
***x* = 0.01**	0.24	0.05	1	1	1
***x* = 0.05**	0.34	0.15	2.8	2.3	0.84
***x* = 0.10**	0.41	0.22	4.1	2.3	0.57

*** relative to *x* = 0.01 sample**

Table S4. Spectral parameters of $Cs_2AgIn_{1-x}Cr_xCl_6$ with x = 0.01, 0.05, and 0.10.

	$^4A_2 \rightarrow {}^4T_1$	$^4A_2 \rightarrow {}^4T_2$	$^4A_2 \rightarrow {}^2E$	*Dq*	*B*	*C*	*Dq*/*B*	*C*/*B*
	cm^{-1} (nm)	cm^{-1} (nm)	cm^{-1} (nm)	cm^{-1}	cm^{-1}	cm^{-1}	-	-
***x* = 0.01**	17 280 (578.7)	12 110 (825.6)	14 350 (697)	1211	529.1	3470	2.29	6.56
***x* = 0.05**	17 160 (582.6)	12 120 (824.8)	14 350 (697)	1212	510.9	3508	2.37	6.86
***x* = 0.10**	17 060 (586.1)	12 060 (829.5)	14 350 (697)	1206	507.3	3516	2.38	6.93

Table S5. Parameters obtained from fitting equations (*eq*7) and (*eq*8) to temperature-dependent emission intensities and decay times of $Cs_2AgIn_{1-x}Cr_xCl_6$ with $x = 0.01$, 0.05, and 0.10.

		$x = 0.01$	$x = 0.05$	$x = 0.10$
$\boldsymbol{A}$ (a. u.)		0.84 ± 0.12	1.03 ± 0.14	1.10 ± 0.18
$\boldsymbol{\tau_0}$ (µs)		26.63 ± 0.25		
$\boldsymbol{\hbar\omega}$ (cm^{-1})		430 ± 80		
E_{nr} (cm^{-1})		3030 ± 90		
s (10^{12} s^{-1})		2 ± 1		
E_{et} (cm^{-1})		253 ± 8		
p (10^4 s^{-1})	PL int	−1.8 ± 4	8 ± 7	25 ± 13
	decay	2.9 ± 1.5	9.0 ± 1.6	19 ± 2

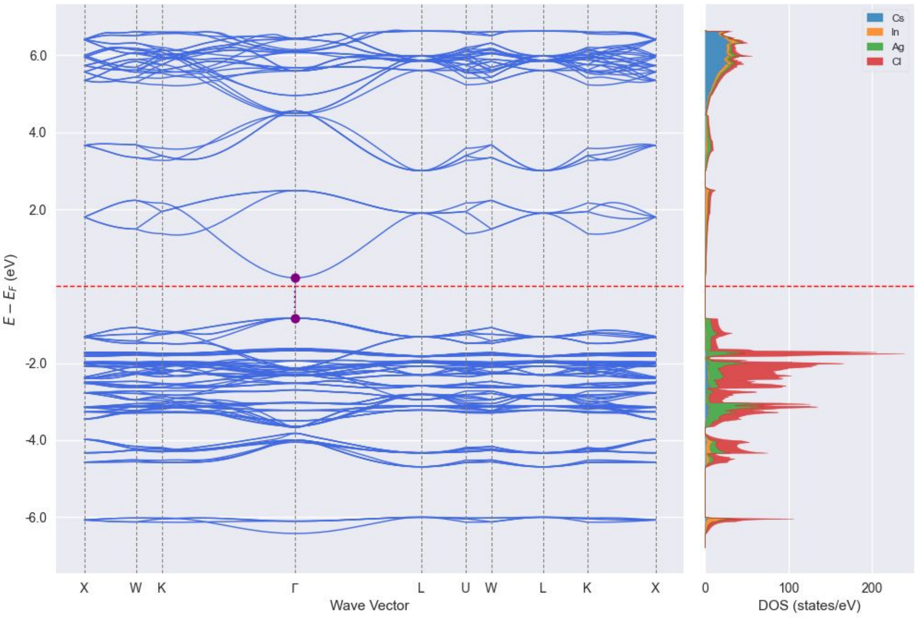

Figure S5. DFT-calculated energy structure and stacked projected density of states of elements in the $Cs_2AgInCl_6$ host.

References:

(1) Giannozzi, P.; Andreussi, O.; Brumme, T.; Bunau, O.; Nardelli, M. B.; Calandra, M.; Car, R.; Cavazzoni, C.; Ceresoli, D.; Cococcioni, M. Advanced Capabilities for Materials Modelling with Quantum ESPRESSO. *J. Phys.: Condens. Matter* **2017**, *29*,

465901.

(2) Giannozzi, P.; Baroni, S.; Bonini, N.; Calandra, M.; Car, R.; Cavazzoni, C.; Ceresoli, D.; Chiarotti, G. L.; Cococcioni, M.; Dabo, I. QUANTUM ESPRESSO: A Modular and Open-Source Software Project for Quantumsimulations of Materials. *J. Phys.: Condens. Matter* **2009**, *21*, 395502.

(3) Garrity, K. F.; Bennett, J. W.; Rabe, K. M.; Vanderbilt, D. Pseudopotentials for High-Throughput DFT Calculations. *Comput. Mater. Sci.* **2014**, *81*, 446–452.
